# Post Total Thyroidectomy Deglutition Syncope

**DOI:** 10.7759/cureus.32836

**Published:** 2022-12-22

**Authors:** Mohamed Jailani, Mahmoud Barbarawi, Supriya Tigadi

**Affiliations:** 1 Internal Medicine, University of Connecticut, Farmington, USA; 2 Pat and Jim Calhoun Cardiology Center, University of Connecticut, Farmington, USA

**Keywords:** paroxysmal av block, situational syncope, sinus pause, post-thyroidectomy complications, dual chamber pacemakers, deglutition syncope

## Abstract

Deglutition syncope is a form of situational syncope where patients develop presyncope or syncope during swallowing. This condition has been observed to occur most commonly in patients with prior gastroesophageal conditions. Our patient developed deglutition syncope that started to occur a few weeks after undergoing a total thyroidectomy. The patient was found to have paroxysmal atrioventricular (AV) block, with up to 5.1 seconds of asystole during swallowing, manifested with episodes of dizziness and lightheadedness. A barium swallow test revealed normal peristalsis and no evidence of dysmotility. The patient underwent placement of a dual chamber pacemaker, and the syncopal episodes resolved. Interrogation of the pacemaker showed no recorded abnormal events.

## Introduction

Deglutition syncope is a form of situational syncope, which is a type of reflex-mediated syncope. Patients generally develop presyncope or syncope during swallowing. The mechanism has not been fully explained, nor understood. However, it is thought to be due to aberrant vagal stimulation [1]. This condition occurs most commonly in males, but its prevalence cannot be estimated [2]. In this case report, we present a patient with deglutition syncope after total thyroidectomy.

## Case presentation

The patient is a 64-year-old female who presented to the emergency department complaining of dizziness and lightheadedness episodes which have occurred over several months. The patient had a past medical history of a non-ST elevation myocardial infarction, diabetes mellitus, hypertension, gastroesophageal reflux disease managed with a proton pump inhibitor, coronary artery bypass grafting procedure in 2020 and hyperthyroidism for which she underwent an uncomplicated total thyroidectomy in early 2022.

A few weeks after the total thyroidectomy procedure, the patient was evaluated for brief episodes of dizziness and lightheadedness that often occur while eating. The patient underwent a barium swallowing procedure which revealed a normal initiation of swallowing, normal esophagus with normal peristalsis, no evidence of strictures, and no hiatal hernia.

Further follow-ups with the use of a Holter monitor revealed frequent episodes of sinus pauses during swallowing of both soft and hard foods. Accordingly, the patient was diagnosed with deglutition syncope. The patient was given multiple therapeutic options of either placing a pacemaker, surgery or prescribing a selective serotonin reuptake inhibitor. The patient was prescribed Sertraline and later underwent another Holter monitoring assessment. Review of tracings revealed repeated episodes of sinus pauses occurring during swallowing. During these episodes, the patient experienced transient dizziness which last for a few seconds, however, returning to her baseline very rapidly.

**Figure 1:**
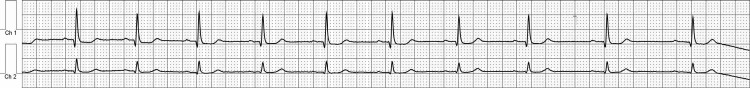
Normal sinus rhythm prior to the episode

**Figure 2:**
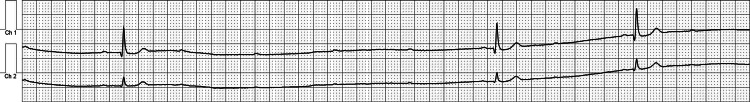
Paroxysmal atrioventricular (AV) block, with up to 5.1 seconds of asystole

**Figure 3:**
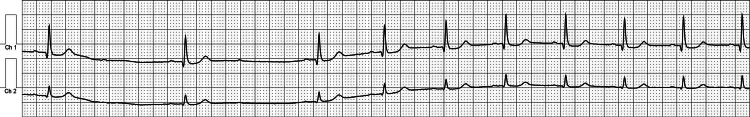
Continuation of paroxysmal atrioventricular (AV) block, followed by normal sinus rhythm

During this assessment, the patient was at home eating and began to experience the same symptoms. Promptly, the patient was alerted that her heart went into episodes of paroxysmal atrioventricular (AV) block, with up to 5.1 seconds of asystole and was advised to visit the emergency department (Figures 1-3).

Upon examination, the patient was alert and oriented with no focal neurological deficits. The patient had a regular heart rate and rhythm, with normal cardiovascular and pulmonological auscultation. An electrocardiogram (EKG) done at this visit showed normal sinus rhythm with no ST segment of T wave changes. There was no evidence of any AV blocks. Telemetry showed similar monitoring findings. The patient was advised to undergo pacemaker placement. The procedure was uneventful, and the patient was discharged after receiving a dual chamber pacemaker. In the outpatient clinic, the patient was followed up and the syncopal episodes had resolved. Accordingly, the pacemaker was interrogated. The dual chamber pacemaker in the dual chamber pacing (DDD) mode was functioning well with no abnormal events.

## Discussion

Deglutition syncope is associated with the occurrence of several gastroesophageal conditions such as gastroesophageal reflux disease. Other conditions include esophageal web, stricture, achalasia, hiatal hernia, and Schatzki’s ring [2-6]. In our patient, a barium swallow did not reveal any abnormalities. However, the patient complains of gastroesophageal reflux episodes, and was controlled with a proton pump inhibitor. A case in the literature describes the occurrence of asystole during swallowing in a patient with hypoxia after an aortocoronary bypass surgical procedure, which resolved spontaneously after normalization of gas exchange [7]. Our patient has a history of coronary artery disease for which she was managed with a coronary artery bypass grafting surgical procedure. She did report good functional capacity without symptoms and her EKG did not reveal any abnormalities [7]. Of note, there was a temporal association between her episodes of dizziness and lightheadedness and the total thyroidectomy procedure. These symptoms started to appear a few weeks after the total thyroidectomy and had never complained of such symptoms prior to the procedure. Possible complications of a total thyroidectomy are the appearance or the worsening of dysphagia, decrease in upper esophageal pressure, and upper esophageal incoordination [8, 9]. However, our patient was evaluated for such abnormalities, and none were found. The potential association between thyroidectomy and deglutition syncope has not been documented in the literature thus far. Our patient did have a large-sized thyroid gland prior to the surgery, which might have led to structural changes within the esophagus that ultimately led to overstimulation of the vagus nerve upon swallowing, causing these sinus pauses. Another potential cause would be a vagus nerve injury during the total thyroidectomy procedure. More data and studies into this condition are warranted to fully understand the mechanism behind these episodes.

This condition is generally diagnosed by obtaining a thorough history from the patient. Identifying the most common triggers to such episodes of pre-syncope or syncope is vital in arriving at this diagnosis. In our case, the patient had noticed the development of symptoms during eating. Holter monitor confirmed this with the presence of asystole with a 5.1-second sinus pause during eating.

Some previously identified common triggers are carbonated beverages, cold beverages, sandwiches, and sticky foods [3, 10-13]. One of the methods of managing this condition is to identify and eliminate triggers. However, our patient developed symptoms to random triggers that could not be easily identified and eliminated.

Other methods of management include the withdrawal of medications that can reduce cardiac conduction such as beta blockers and calcium channel blockers [2, 3]. Our patient had not been taking such medications. Accordingly, the best approach was to place the patient on a dual-chamber pacemaker as was observed in previous reports [3, 14].

## Conclusions

Deglutition syncope is a form of situational syncope and has been observed to occur in patients with prior gastroesophageal conditions. Our patient developed deglutition syncope after undergoing a total thyroidectomy, and the syncopal episodes had resolved after the placement of a dual-chamber pacemaker. This occurrence has not been documented previously in the literature.
